# Quality Improvement in General Dental Practice: Situational Analysis for the United Kingdom and Germany

**DOI:** 10.1177/23800844241306734

**Published:** 2025-02-23

**Authors:** C. Lin, V. Fehrer, L. O’Malley, W. Thompson, S. Listl, M. Lorenz, M. Byrne

**Affiliations:** 1 Division of Dentistry, University of Manchester, Manchester, UK; 2 Section for Oral Health, Heidelberg Institute of Global Health, Heidelberg University Hospital (UKHD), Heidelberg, Germany

**Keywords:** oral health, dental care, dental care team, quality of health care, quality indicators, qualitative research

## Abstract

**Introduction:**

Quality improvement (QI) is important in ensuring standards in oral health care. Despite the growing literature on quality indicators, audit, and feedback, the perceptions and expectations of oral health professionals toward QI remain unclear. Understanding these perspectives, barriers, and facilitators is important to effectively encourage and maintain QI activities in dental practices. This is the first of 6 publications in a series titled “Situational Analysis of QI in Oral Health Care in Europe.”

**Aim:**

This study aimed to investigate how QI was conducted and perceived in dental practices in the United Kingdom and Germany.

**Methods:**

A situational analysis, comprising desk research and semistructured interviews, was conducted. Data collection was conducted from May to October 2023. Purposive and snowball sampling techniques were used to recruit dental practice participants in the United Kingdom and Germany. Interviews and key texts were thematically analyzed to synthesize 3 maps: a situational map, a social world map, and a positional map.

**Results:**

Eighteen participants were interviewed, comprising dentists, dental hygienists, dental therapists, dental nurses, and practice managers. The participants described 6 competing positions surrounding QI: QI activities were expressed as being worthwhile or box-ticking exercises to satisfy regulators. Some felt that QI detracted from service delivery and should not be the role of the dentist, whereas others stressed the need for a whole-team approach. Some felt that patients were important to judge quality, whereas others felt quality in dentistry required understanding of technical processes beyond the reach of patients.

**Conclusion:**

This study provided insights into how QI activities were carried out in dental practices in the United Kingdom and Germany and how it is was perceived by those working in this environment. This study offers key observations into the situations, social worlds and arenas, and positions that influence QI in dental practices.

**Knowledge Transfer Statement:**

The findings from this study highlight several contextual barriers and facilitators to quality improvement in general dental practice. Understanding these determinants of quality improvement is relevant for oral health teams and dental practice managers who aim to develop and implement quality improvement strategies in dental practice.

## Introduction

Quality improvement (QI) is a central part of the clinical governance of health systems (Macfarlane 2019). QI is a cyclical process in which current standards of care are continuously measured to assess current performance, inform changes in practices, and improve standards. The previously suggested definition of quality of the Institute of Medicine refers to safety, effectiveness, efficiency, equity, timeliness, and patient-centeredness as important domains of health care quality (Institute of Medicine Committee on Quality of Health Care in America 2001).

QI in oral health care can have many purposes, such as stimulating behaviour change in clinicians, informing funding and purchasing decisions, and rewarding good performance (Campbell and Tickle 2013; Tickle and Campbell 2013). QI activities could bring various benefits to the practices, including improved patient satisfaction, personal and organizational development, and business outcomes (Crisan et al. 2021).

QI allows a practice to identify where their performance is good and where it can be improved (Backhouse and Ogunlayi 2020). However, if quality is only assessed, and these assessments are not acted upon by changes in structures, processes, and the behaviors of staff, quality is unlikely to improve (Backhouse and Ogunlayi 2020).

At the clinician level, translation of quality assessment into QI can be conceptualized using the Clinical Performance Feedback Intervention Theory (Brown et al. 2019). This states that a clinician’s intentions and subsequent behaviors to improve clinical performance are informed by reflections upon their perceived performance and formalized feedback. Meaningful changes in actions and behaviors are easier to make when feedback is targeted, personalized, and specific (Baâdoudi et al. 2020; Rosing et al. 2020).

QI activities in dental practice are considered as either mandatory or voluntary arrangements (Baâdoudi et al. 2016; Baâdoudi et al. 2019; Berwick 2016). Mandatory arrangements are typically “top-down” arrangements, imposed by funders, insurers, and regulators to demonstrate that practices are meeting a “minimum” standard (Campbell et al. 2001; Patel and Jenkyn 2021; Care Quality Commission 2023). These arrangements may include mandatory audits of accessibility, decontamination of instruments, radiographic images, or inspections from regulatory bodies (Patel and Jenkyn 2021; Care Quality Commission 2023). There is a legal obligation to comply with these processes of quality assessment and improvement (Care Quality Commission 2023). Previous literature has suggested that mandatory QI can be viewed as paternalistic and misguided, resulting in feelings of suspicion, resistance, and helplessness among health care professionals. (Baâdoudi et al. 2016; Berwick 2016; Baâdoudi et al. 2019).

Voluntary arrangements by contrast are typically “bottom-up” approaches to quality assessment, in which QI techniques are used to drive reflection and behavior change in an individual or a team in the practice setting. This may include personal or local audits, continuing professional development, or the use of practice accreditation schemes, such as the British Dental Association Good Practice Scheme in the UK (British Dental Association 2023). Voluntary arrangements in Germany include “Quality Circles” endorsed by the state chambers of dentists in which groups of dentists meet regularly to discuss quality in dental practice (Landeszahnärztekammer Baden-Württemberg and Körperschaft des öffentlichen Rechts 2015). Quality circles are characterized by a systematic and targeted approach, a time limit, and a host who moderates the sessions, supports the autonomy of the group, provides assistance in solving problems, and ensures a group atmosphere that allows for equal discussion among the participants.

Both the United Kingdom and Germany have well-established oral health care systems that provide high-quality care to their populations based on mixed public and private oral care services (Ziller et al. 2015; Gulabivala 2018). Both countries have established regulators, namely, the General Dental Council in the United Kingdom and the Federal Mistry of Health in Germany, and national bodies, such as the Care Quality Commission (CQC) for the United Kingdom and the German Dentists Association (Bundeszahnärztekammer) to regulate the quality of the services in dental practices.

Despite these similarities, some differences in the structure of services and funding also emerged. The per capita expenditure on dental health care in 2019 has been reported to be lower in the United Kingdom (USD 143.2) than in Germany (USD 372.2) (Jevdjevic and Listl 2024). Each nation in the United Kingdom operates under a slightly different National Health Service (NHS) contract. For example, in England, practices contracting with the NHS receive payment based on a banded fee structure (a fixed fee, regardless of the number of procedures within that band). However, in Scotland, practices are paid on a fee-per-item basis (a separate payment for each specific treatment). In Germany, there is a wide range of different statutory health and private health insurers, existing in in a federated system. In addition, in the United Kingdom, the NHS provides subsidized basic and preventive dental treatments, with no provision for cosmetic work and limited access to advanced care in the general practice setting (Gulabivala 2018). On the other hand, in Germany, a mixed system of statutory health insurance (Gesetzliche Krankenversicherung) and private health insurance (Private Krankenversicherung) provides oral health services (Ziller et al. 2015). While the statutory health insurance offers a generous and comprehensive coverage compared with other countries, specialized and esthetic treatments are covered only by private insurance. The statutory health insurance is funded equally by employees and their employers and focuses on basic and essential dental treatments. In contrast, private health insurance offers comprehensive health services for those who opt out of statutory health insurance (e.g., self-employed individuals). For those who remain within the statutory health system, private health care schemes can provide top-up coverage for oral care, usually for specialized and esthetic treatments (Ziller et al. 2015). Given the similarities and differences between the 2 countries, this study could gain deeper insights into how well-established but distinct oral care systems respond to dental practice QI.

The situational analysis method evolved from traditional grounded theory to explore social phenomena in complex environments (Clarke 2003; Clarke et al. 2018). In grounded theory, a social world is identified as a group of people who have collective patterns of interaction, meanings, and practices (Clarke 2003; Clarke et al. 2018). This method examines how a health professionals’ social world, and the knowledge inherent to this world, have developed, evolved, and overlapped with other arenas over time. By mapping the situations, social worlds, and positions held in areas of interest, situational analysis may be used to understand the cultural, economic, historical, and social positions that have led to knowledge and beliefs (Clarke 2005).

QI exists in a complex social and professional arena. A number of cultural and contextual factors affect how QI is approached, enacted, and perceived by members of the dental profession. The dental practice is the most common setting in which oral care is received; however, this is a setting with wide variations in cultures, contexts, and financing methods. The comparatively siloed nature of oral care means that QI is less well established than in other fields of health care. There is little discussion in the academic literature about the impact that social worlds and positions that arise from the dental practice setting may have on QI. Situational analysis is a useful tool in which researchers can examine the complex situations and phenomena associated with a health care setting such as the dental practice.

QI in oral health care can have various forms to different stakeholders. To improve quality, sound methods for QI and behavior change must be considered. The DELIVER Project is a European Consortium project funded by Horizon EU that aims to deliver actionable knowledge on best practices to improve quality in oral health care. This is 1 of 6 publications in a series titled “Situational Analysis of QI in Oral Health Care in Europe: From Stakeholder Perspective to Action.” The articles in this series aimed to demonstrate the current situation of QI in the United Kingdom and European Union on practice level, community level, country level policy level, and EU/global policy level. This study uses situational analysis methods to identify the key actors, social arenas, and positions of stakeholders that influence how QI is conducted and perceived in primary oral health care, using desk research of key texts and exploratory interviews. The foci included the interactions and performance of individual practitioners, the practice as an organization, and the different approaches practices take in providing quality service for their distinct target populations of patients. In addition, it covered management and oversight activities, clinical input, facilitators, and barriers to implementing QI as discrete practice entities.

## Methods

### Aims

The aim of this study was to address the following questions at the practice level in the United Kingdom and Germany:

- What are the existing QI activities being undertaken?
- Who are the stakeholders involved in QI?
- Which stakeholders have influencing power in QI and what are the relations between these stakeholders?

### Literature Review and Desk Research

Prior to primary data collection, initial elements for a situational map and a social world map were discussed in both the United Kingdom and Germany contexts within the research team (C.L., V.F., M.L., and M.B.). The authors then drafted a situational map and a social world map. The situational map outlined the known actors involved in QI, their relations and paths of communication, and the underlying principles of QI. This mapping exercise informed focused literature searches of the websites of key informants to understand what resources and literature were available around QI in oral care (C.L., V.F., M.L., and M.B.). This was supplemented by searching PubMed and Google Scholar with keywords such as “quality improvement” and “dental care/oral care” in April and May 2023. Known quality (improvement) programs in the respective countries and the legal anchoring of quality in the respective health care systems were also identified. In these steps, key elements of QI in oral health care were gathered to refine the maps and were used to develop interview questions and inform the situational analysis later.

### Qualitative Interviews

Qualitative interviews were conducted by the 2 health services researchers (C.L. and V.F.) to assess the key elements from the literature review and desk research and to determine how QI is performed and perceived by oral health professionals. These interviews were semi-structured in nature, and the interview guide was developed based on the findings of the desk research and the Consolidated Framework for Implementation Research (Damschroder et al. 2022). The interview guide covered what approaches to quality assessment were currently being used in the United Kingdom and Germany and how this translated into QI activity. The main topics included understanding and definition of QI, tension for change, relative priority, efforts, external policies and incentives, patients’ needs and resources, and learning climate. The Medical Faculty Heidelberg Ethics Committee (reference S-089/2023) and the University of Manchester Ethics Committee (reference 2023-16459-28568) reviewed this study, and approval was granted.

### Participants and Settings

Participants were identified by purposive and snowballing sampling techniques. The inclusion criteria were that participants had to be oral health professionals including dentists, dental hygienists, dental therapists and dental nurses, and practice managers. Participants were selected from the general dental practice environment in the United Kingdom and Germany. These participants were selected to better understand the situation and positions of the general dental practice setting and how QI activities are planned, carried out, and acted upon.

### Data Collection

Recruitment advertising for interviews was disseminated through existing research contacts, and through collaboration with professional bodies including the British Dental Association, Care Quality Commission, Association of Dental Groups, College of General Dentistry, the German Society for Dental Hygienists, and Akademie für Zahnärztliche Fortbildung Karlsruhe. To ensure a maximum variation sampling approach, potential participants from different sectors and sizes of dental practices (such as state, private, or mixed; small/large; and corporate/noncorporate practices) were invited to participate. For Germany, potential participants from each federal state were invited to gain sufficient insight, as regulations and QI activities may vary between the federal states. The participants were provided with an information sheet, and signed informed consent forms were obtained from the participants before the interviews.

In-depth semistructured interviews with an interview guide were used to explore participants’ perspectives on the topic. No modifications were introduced to the interview guide. Interviews were conducted by one of the first authors (C.L. for interviews in the United Kingdom; V.F. for interviews in Germany). C.L. is a female health service researcher and a registered nurse who is well trained in qualitative interviewing techniques. V.F. is a female doctoral candidate, speech-language therapist, and trained in interprofessional health care and health services research and implementation science. Interviews were conducted online, by telephone, or in person, digitally recorded and transcribed verbatim. Alongside the interviews, field notes were taken by the interviewers. To ensure data protection, a data management plan was reviewed and approved by the ethics committees. In this study, to reach the data saturation point, data collection continued until no new codes or themes were generated from additional data (Tran et al. 2016). Through regular meetings between the first authors (C.L. and V.F.) during data collection and analysis, the richness of the data was ensured. Saturation of data, when no new codes or themes were generated from the interviews, was achieved after interview 10 in the United Kingdom and interview 6 in Germany. Additional interviews were conducted to ensure this finding.

### Data analysis

The situational analysis in this study focused on the context of the general dental practice setting within the United Kingdom and Germany. The aim of this method was to generate explicit maps of contextual situations (including persons, objects, or event factors; Clarke 2003; Clarke et al. 2018). Three maps, namely, a situational map, a social world map, and a positional map, were generated (Clarke 2003; Clarke et al. 2018).

**Figure 1.**
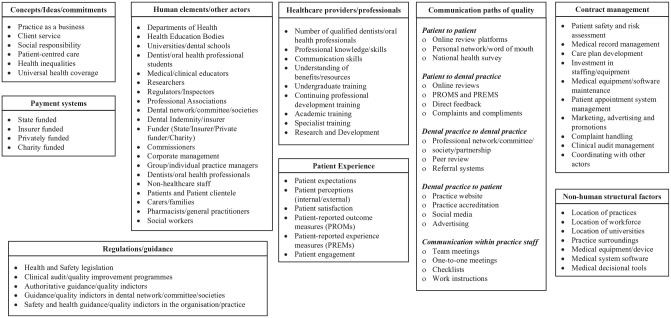
Situational map.

Situational maps report key contextual elements (human, nonhuman, and discursive elements) that explore a phenomenon and examine the relationships between elements. The mapping process started with a “messy map” (not shown here) that is created by describing all human and nonhuman elements, who and what is in this situation, who and what is important in this situation, and which elements make a difference in this situation. These descriptions are written down randomly and “messy” so that the team can focus on the ideas and not have to deal with the structure at this stage. This map is not a final version but is intended as a starting point to stimulate thinking and recognize patterns and connections in the data. Derived from this messy map is an “ordered map” referred to as a situational map (Fig. 1). Once the important elements are put down on paper, they are grouped or categorized to make sense of the topic. Clarke suggests 12 headings for the ordered map but encourages the researcher to use headings that make sense for understanding the data (Clarke 2003).

Social world maps aim to explore how the elements are situated in social arenas around the circumstance. Clarke et al. (2018) and Clarke (2003) described a social world as a collective pattern of interaction, meanings, and practices within a group of people defined by their common interests or concerns; a social arena is a space where multiple social worlds converge. Thus, within an arena there will be social worlds that pursue different objectives, influencing the outcomes of the social arena. Finally, positional maps present particular positions taken and not taken where the phenomenon occurs and identify silent positions. Thematic analysis was performed with NVivo software (version 12) in the United Kingdom and MAXQDA Analytics Pro 2022 in Germany. Open, axial, and selective coding were applied iteratively according to grounded theory (LaRossa 2005). The analysis started with line-by-line coding, and a list of initial codes emerged. To proceed in a more focused manner, the authors then identified and categorized these codes into selective codes relevant to the research question. Finally, the categories were evaluated, and a set of core categories was identified. The 2 first authors had regular meetings and were actively involved in the coding process to reach an arrangement of codes and themes and to ensure they represented the full dataset.

### Rigor

Lincoln and Guba proposed 4 principles of rigor—credibility, transferability, dependability, and confirmability—which underpin study quality (Lincoln and Guba 1985). Several strategies were used in this study to achieve these goals. Peer checking meant obtaining advice from other academic colleagues to ensure credibility and dependability. To enhance transferability, a detailed and thick description of the study process and methods is provided. An audit trail of all stages and procedures of the study was also documented to ensure confirmability, which can be made available through contacting the first authors.

## Results

### Characteristics of Participants

**Figure 2.**
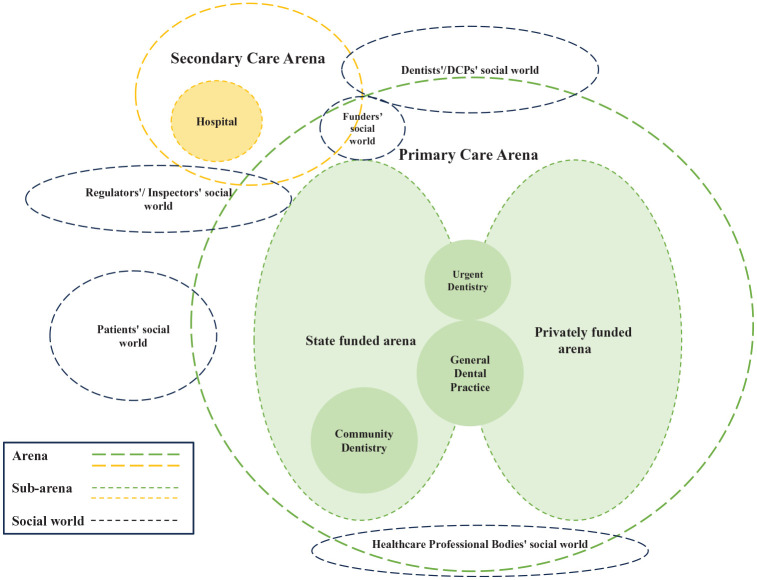
Social world map of arenas in general dental practice.

**Figure 3.**
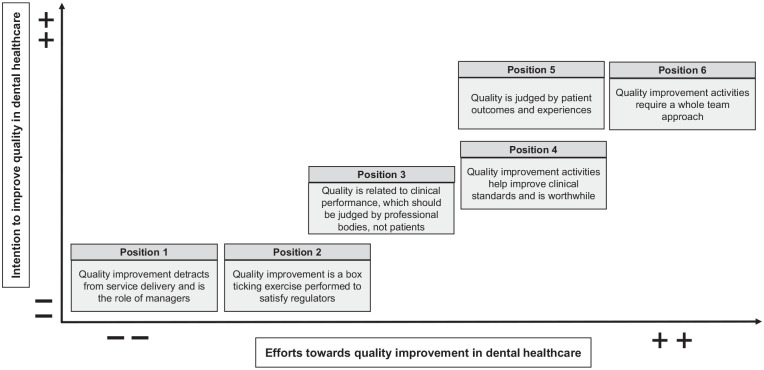
Positional map.

The interviews were conducted between May 2023 and October 2023. Eighteen participants (11 in the United Kingdom and 7 in Germany) who were working in general dental practices agreed to be interviewed and then took part in the interviews. Eleven dentists, 4 dental hygienists, 1 dental nurse, and 2 practice managers participated this study. The participants comprised 6 men and 12 women with an average age of 45.3 y (range from 29 to 62 y old, median = 44 y old; 1 participant did not disclose age information). The interview duration ranged from 23 min to 52 min, with an average of 37 min. Three maps were generated: a situational map (Fig. 1), a social world map (Fig. 2), and a positional map (Fig. 3).

### Situational Map

Figure 1 presents important contributory factors and elements that influence QI in general dental practices, as identified from the literature and interviews. The key concepts that were discussed and identified as underpinning elements of QI activities were the competing nature of patient-centered care and the need to run a practice as a profitable business. There was a strong connection between the time and resources available and different payment systems as well as a perceived influence of different payment systems, regulations, and guidance on current efforts to improve quality. The importance of human elements and actors was well recognized in the interviews. For example, key stakeholders and sectors such as government departments, medical education bodies, health professional bodies, funders, health professionals, other staff, and patients played various roles in QI activities. Furthermore, nonhuman structural factors, such as the location of practices, location of workforce, and practice surroundings, were identified as fundamental to improving the quality of oral health care.

Some in-depth discussions highlighted that patient factors and health professional factors contributed to QI activities. The participants also pointed out that duties regarding contract management could be key to QI activities in the practices. Participants also believed that several communication paths with different sectors as well as within the dental practice should be considered for QI in general dental practices.

### Social World Map

Figure 2 represents the social worlds and arenas where QI activities take place in dentistry. Social worlds are groups of people defined by their common goals, interests, or concerns. Arenas are spaces or contexts in which different social worlds come together. In the map, several social worlds were identified: dentists’/Dental Care Professionals social world, patients’ social world, regulators’/ inspectors’ social world, health care professional bodies’ social world, and funders’ social world. The main arenas identified were the primary care arena (subarenas: state-funded arena and privately funded arena, general dental practice, urgent dentistry, and community dentistry) and secondary care arena (subarenas: hospital).

General dental practices were captured within primary oral health care, but a practitioner’s social world also extended into secondary care due their role as a referrer and gatekeeper to secondary care (Starfield et al. 2005). Within the primary dental care arena there are 2 subarenas, namely, state funded in the United Kingdom and statutory health insurance based in Germany and privately funded, which create various social worlds for the stakeholders. Patients’ social world actively participates in the primary care arena to be a part of QI activities. By contrast, dentists’ and oral health professionals’ social world is significantly active in both primary and secondary social arenas to contribute to debating, negotiating, and coordinating the QI activities for oral health care. In addition, regulators’/inspectors’ social worlds span both primary and secondary arenas to ensure strategies and policy on QI are well identified. Within funders’ social world, they would be active in the primary and secondary care arenas where funding and resources for QI are allocated. Finally, the social world of health care professional bodies lies at the bottom of the primary care arena, developing and generating knowledge relevant to QI.

### Positional Map

The positional map (Fig. 3) represents different positions taken in the discourse on key issues raised in the interviews. In Figure 3, the vertical axis shows intention to improve quality in oral health care, and the horizontal axis indicates efforts toward QI in oral health care. Although some efforts had been made to improve quality in dental practices, various challenges had also emerged. There was a significant absence of (1) positions very high in intention and low in effort and of (2) positions very low in intention and high in effort. The following 6 positions were identified from the interviews:

- Position 1: QI detracts from service delivery and is the role of managers.
- Position 2: QI is a box ticking exercise performed to satisfy regulators.
- Position 3: Quality is related to clinical performance, which should be judged by professional bodies, not patients.
- Position 4: QI activities help improve clinical standards and are worthwhile.
- Position 5: Quality is judged by patient outcomes and experiences.
- Position 6: QI activities require a whole-team approach.

### Position 1: QI Detracts from Service Delivery and Is the Role of Managers

Overwhelmingly, all participants felt that heavy workloads and limited time could be key barriers to engaging in the QI activities. These relegated QI to not be seen as a priority; it made it challenging, requiring more effort to undertake it. In the United Kingdom, this could be particularly relevant for state-funded practices, where the challenges are even greater, as illustrated by this quote:

I would say time and just the volume of paperwork, particularly with the NHS [National Health Service in the United Kingdom] stuff, you know, there’s so much, it just is another thing that gets shoved away, unless you have to do it, there’s so much you have to do. Yeah, I think it just gets prioritized, you know, the minimum is done. (R10)

A strong sense that QI requires time and effort emerged in the interviews. Most participants felt that members of the oral health team, even if they were aware of the importance of QI, were under too much time pressure to take further action about it:

We can’t stop treating patients and only do quality management, we have to have time for the treatment and when the treatment is finished, and the documentation has to be done, and the accounting has to be done, and many other things have to be done, and the patients want to be well cared for. (Int_3)

Regarding time requirement for QI activities, the oral health professional participants believed that QI activities needed “someone else” to fully engage. Generally, practice managers and administrative assistants were identified as the people who should take on these duties, in the participants’ opinions.

She [dental assistant] has put everything in folders in a very old-school way, and we constantly monitor whether we are doing the right thing. For example, there are always forms that have to be updated regularly, that have to be signed, instructions on certain topics, etc., and she makes sure that everything is always up to date and always reads through the guidelines if they should change or something. (Int_2)

Due to the nature of the payment system in the practices, practitioners’ income was normally related to clinical time. Efforts regarding QI were sometimes underpaid or unpaid. The interviews show that oral health professionals therefore make little or no efforts to improve quality. This was highlighted by a participant in Scotland, where it was described that previously mandated audit activity was being omitted when contractual obligations to conduct audits were removed:

All NHS [National Health Service in the United Kingdom] dentists in Scotland have to do these 15 h [of quality improvement activities] over the 3 y. And it used to just be that you would do an audit on your LDU [local decontamination unit], you would do an audit on handwashing, you’d do an audit on your radiographic quality and things like that. But our chief dental officer has put a pause on that just now. So, nobody’s doing any kind of audit or quality improvement projects at the moment until that’s all brought back in. (R03)

### Position 2: QI Is a Box-Ticking Exercise Performed to Satisfy Regulators

Almost all participants recognized that meeting the minimum standards of regulators was an important end. Given the importance ascribed to mandatory audits, participants felt that successful completion of audits was the most significant evidence of quality in their practices:

Oh, so we do have regular audits of dental records and radiographs and things like that, no we do that every . . . whatever the requirement is. I’ll get an audit sheet to do, and I’ll do it. (R02)

Despite reasonable efforts to audit for QI, some participants believed that clinical audits and quality management were performed perfunctorily and with resignation. The issue of no subsequent action being taken after the audits was raised by the participants. These audits were seen as a box-ticking exercise rather than an opportunity to assess and improve the quality of care:

All the audits produced by the major compliance companies and by the BDA [British Dental Association], and by Denplan, are just basically tick boxes. There isn’t a . . . and it’ll just say “yes,” or “no,” tick. (R11)

### Position 3: Quality Is Related to Clinical Performance, Which Should Be Judged by Professional Bodies, Not Patients

In the interviews, some questions such as “how should the members of the oral health team measure their performance and outcomes?” and “what and whom should they measure?” were also highlighted. Participants described clinical performance in dental practices as a key indicator of QI. The importance of routine externally and internally reported quality assessments in health care was also widely recognized. Participants felt that QI needed to cover a wide range of aspects of clinical performance, such as infection prevention and control, antimicrobial prescribing, and clinical record keeping. Most participants believed that these should be judged by the technical standards of health professional bodies. Positive feedback from these bodies could encourage health professionals to make efforts to undertake QI activities:

Well, the CQC [Care Quality Commission] will have 4 criteria to enforce and if you’re deficient, at the bottom of the scale, you can be closed down and [get] lots of enforcement notices. So, and at the higher end of, you know, if everything is satisfactory, they just tell you that you’re satisfactory. And if you are really good, they will issue a “notable practice” note, to say they’ve found something relating to patient care which is innovative and exemplary. And they will publish that. (R04)

Participants emphasized the quality of oral health care in terms of the clinical focus, judged by dentists and health professionals. The participants thought that patients without professional and clinical knowledge were incapable of assessing the quality of oral health care:

You can ask the patient how they got on—broadly speaking, a useless exercise in my opinion because they are unable to judge the clinical quality of what’s been done. (R08)

Although the importance of patient experience and views has been widely accepted, participants felt that it would be difficult for patients to assess clinical performance and treatment outcomes. Another participant pointed out that patients and oral health professionals had different focuses:

The other thing is patients aren’t necessarily aware of the quality of their treatments. They’re aware of how long they wait, they’re aware if they’re in pain, they’re aware if somebody was nice to them, but they don’t know how good my filling is. So yeah, that’s the other thing from a patient side of view, is, their perception of quality and my perception of quality are markedly different. (R10)

### Position 4: QI Activities Help to Improve Clinical Standards and Are Worthwhile

Participants also highlighted the idea that even experienced health professionals might make medical errors and mistakes in oral health care settings. It is obvious that these mistakes could cause risk and harm for patients in oral health care. To preserve the safety and health of patients, health professionals must make concerted efforts to avoid medical errors and mistakes. This sense that “we’re not perfect” motivated the oral health team to engage with high standards of care quality:

We are not perfect, and we do make mistakes, and we try and minimize the mistakes and learn from them, and I guess that is what people say is improving quality. (R04)

The participants also expressed their view that self-reflecting on performance in dental practices was a critical way for the members of the oral health team to examine, review, and learn from their own experiences and actions. They felt that it could then help the oral health team to assess oral health care quality and to be aware of the need to take actions for QI. Such awareness could then motivate them to actively engage in QI activities:

And so, we looked at this and we looked at a number of cases that each dentist had done, and one particular clinician had on multiple cases, too difficult to get the damn crown on, on this tooth . . . et cetera. And it became pretty obvious that the issue wasn’t the tooth or the patient, but the clinician’s inability to use the kit properly. And so further training was required in order to get that clinician up to scratch. (R08)

### Position 5: Quality Is Judged by Patient Outcomes and Experiences

All participants saw patients as a focus of QI in oral health care and felt they should be involved in QI at some level. They believed the main purpose of QI was to meet patients’ health needs. With this belief, the oral health team was more likely to have a positive attitude toward QI and would then be more willing to take further action to improve the quality of oral health care:

Yes, absolutely. I mean, I think, you know, everything that we do is for our patients, to try and improve their journey in the practice, to try and improve the treatment that we’re providing. So, yes, patients are definitely. . . . And I think it’s important to speak to patients. (R03)

Another key factor reported by the participants was focusing on reputation marketing. Some participants (especially project managers) considered their oral health care practice as a commercial business rather than a health care setting. Based on the nature of running a business, some believed they needed to provide high-quality care or “customer service” for patients or “clients” in the dental practices. They make more efforts to determine how to make their clients satisfied with their services and believed this was a major part of QI:

And in the real-world money comes into it and some people have more and are willing to spend more on fixing dental problems than others. So, choices have to be made based on that. Some people can afford a denture, some people can afford implants. So, that is. . . . Clearly the patient’s preference has to be thought [about] and understood so that the dentist can propose the right treatment for the right . . . for that individual. (R08)

### Position 6: QI Activities Require a Whole-Team Approach

The participants, from both private and state-funded practices, reported a sense that they needed to be doing their best to provide high-quality oral health care for the whole community. This required the whole oral health care team to be actively involved in QI activities. The commitment that “we should do the best” as a team for patients was widespread among the participants in the interviews. A few participants paid significant attention to how the oral health team should work as a team to improve the quality of oral health care:

Here in the practice, we have that I don’t do it alone either, but I do it together with the team. It’s just not like that . . . that the practice manager distributes the tasks [laughs]. I got them together and said “okay, we have this and this and this and these tasks, daily, weekly, monthly.” . . . So, don’t decide alone, but say “guys, how can we do this? I’m at the end of my tether, does anyone have a suggestion?” (Int_7)

In addition, the participants believed that the team efforts toward QI could then bring benefits to both oral health professional teams and patients. This could motivate them to put more effort into QI activities:

And once I [as practice manager] complete any audit, we feedback through team meetings, and look at how, as a team, we can improve waiting times, notes, you know, ensuring radiographs are completed, and all the notes are there, and they’re graded et cetera. We discuss that on a regular basis, to ensure that everyone’s complying. Team meetings are really good for feedback like that, and I think having that discussion does remind people that not everyone’s going to be kind, but we need to try harder to ensure that is the only patient that has felt like that. (R06)

Issues such as long waiting times, the backlog in the oral health care system, patients’ failure to attend dental appointments, and the shortage of dentists were highlighted in the interviews as having negative effects on quality oral health care. To tackle those issues, an efficient health care delivery team was also seen as key to ensuring patients received medical treatment in a timely manner and to meet patients’ needs:

Currently we don’t use the whole team well, and I think that it should be a team approach. I think that the patients particularly look towards the wider dental team to implement the strategies. They tend to talk differently and better to the wider team rather than clinicians about their oral health needs. (R07)

## Discussion

This study provided unique and novel insights into the current state of QI in general dental practices. Based on situational analysis in the United Kingdom and Germany, QI is influenced by complex social settings and dynamics, according to views obtained directly from members of the oral health team and review of the literature surround QI.

Overall, the importance of QI has been noted in both this study and the literature (Campbell and Tickle 2013; Tickle and Campbell 2013). Some efforts toward QI have been made in general dental practices (Campbell and Tickle 2013; Tickle and Campbell 2013). For example, an extensive clinical audit scheme and a pilot peer review scheme were introduced in England from 1991 until 2006 (Eaton et al. 1998). However, members of the oral health team engaged at varying levels, ranging from fully engaged to not engaged at all. From the perspective of participants in this study, QI did not yield a position that was very high in intention and low in effort or a position that was very low in intention and high in effort, which could explain the empty areas in the maps. Several challenges were identified in both the literature and this study such as a lack of time and motivation. This suggests that further actions and contractual changes may be necessary to make these initiatives more widely available to general dental practices.

Most participants recognized that QI required a significant amount of time and effort. Both this study and the existing literature found that a heavy workload and limited time were often a major source of complaint from health professional team. Previous studies have also shown that the members of the oral health team were concerned that dental practices were busy, time-poor environments (Doshi et al. 2021). Prioritizing health care to use their time “wisely” could be one strategy for health professionals to manage their time (Butler et al. 2017). Exploring “how to use limited time” could be a facilitator to build interest in QI activities. Some strategies to consider to optimize workflow or QI activities for oral health professionals could be using simpler and more flexible working systems and audit/QI platforms to tackle these challenges. The participants also felt that QI was “someone else’s” duty, meaning someone else in the team was responsible for QI. However, the current literature recommends that QI should not be solely the responsibility of one particular person but all members of the oral health team (Patel and Jenkyn 2021).

The importance of clinical audit as a part of QI has been widely accepted by previous studies (Campbell et al. 2001; Patel and Jenkyn 2021). However, participants in this study did not feel that clinical audits had a significant impact on efficiency, outcomes, or quality of their oral health care. Audits described by the participants were wide ranging, but despite acknowledgment that this required significant time and effort to achieve, they largely depended on only 1 individual to be responsible for conducting and managing clinical audits in a practice. This approach limited the utility of an audit to drive improvements throughout the whole health care team (Boyle and Keep 2018). Furthermore, national mandatory audits require so much data collection that QI resources are limited to performing the audits rather than acting on their results (Boyle and Keep 2018). Therefore, this explained why some members of an oral health team performed quality management activities perfunctorily and with resignation. Further actions and reviews are needed to rethink current clinical audits. Attention should be paid to critical questions such as the topics to include other sources of information to use and how audits could benefit practices and what how other activities could facilitate QI.

Despite some efforts having been made regarding patient involvement in QI, other studies have described patient experience and feedback not being adopted meaningfully or systematically (Gleeson et al. 2016; Marsh et al. 2019). This study found a conflict for the oral health team, that on one hand, quality should be judged by clinical performance and treatment outcome to ensure clinical standards are upheld. Therefore, QI is seen as difficult for patients without any professional knowledge about clinical performance to take part in QI while still knowing that the patient and their experience is a focal point in the dental practice shown as a conflict in the positional map with positions 3 and 5. Recent studies have also found that active patient involvement in QI could provide numerous benefits to health care, such as enhancing interaction between patients and health professionals and improving motivation for behavior change for QI (Bergerum et al. 2019; Bergerum et al. 2020). This suggests the potential and need to better define the role of patients in relation to QI.

Participants’ understanding of QI for oral health care varied in this study. A few participants had limited understanding and desired clearer guidance regarding QI. Understanding requires health professionals to effectively incorporate sufficient knowledge and familiarity with the necessary knowledge to actively engage with and implement clinical activities or interventions (Barth et al. 2016; Almazrou et al. 2020). It is potentially challenging for health professional teams to take further action on QI if they have insufficient information about it. Further actions should be taken to tackle support translation of knowledge into implementation.

Some participants had more positive views about QI supported by studies that show that a positive attitude to QI can significantly increase health professionals’ motivation to engage in QI activities (Butler et al. 2017). Participants in this study perceived that QI was effective and efficient care would be delivered in dental practices (Veenstra et al. 2022).

### Strengths and Limitations

Using situational analysis demonstrated the landscape of QI in general practice in this study. The maps created from the desk research and interviews illustrated various areas that influence QI. The situational map gave a combined overview of human elements as well as health care providers/professionals and nonhuman structural factors and highlighted different paths of communications that need to be considered if quality is to be improved. The social arenas map identified different social worlds of the actors in the health care system, where they overlapped and where they did not to show that the social world of dentists overlaps with many other worlds and arenas. In contrast, the social world of patients did not overlap. This raises the question about whether the world of patients should have more overlap with other actors in the health care system. The positional map indicated various positions that are being taking in the discourse of QI that may be conflicting. Interestingly, the positions are either very low or very high on both axes. The participants in this study showed that their positions aligned with the discourse of the intention to improve quality and the efforts toward QI in oral health care in a positive or negative way.

The limitations of this study should be carefully considered. Although qualitative studies do not aim to be generalizable, the small number of participants (*n* = 18) in this study needs to be taken into account. In addition, patient views were not included in this study, while a varied sample from the health care group (dentists, dental hygienists, dental therapists, dental nurses, and practice managers) was recruited. As the participants were recruited from only 2 countries (the United Kingdom and Germany), it is difficult to draw a firm conclusion for all EU countries. Therefore, further research with patients and various countries could be considered to ensure transferability of findings to different contexts. Despite the limitations, these findings still identified critical themes that could be an important resource to develop effective strategies for QI and further studies on the topic.

## Conclusion

This study provided insights into QI and the key elements from the situations, social arenas, social worlds, and positions identified in the study. Although some dentists and practice managers valued QI activities and some QI efforts have been made in dental care, QI activities are still not seen as being a norm for all members of the oral health team. There is still a mindset that QI is done out of obligation. Several challenges were identified. The idea that QI activities required a significant amount of effort and time was widely accepted. Furthermore, the study showed that negative perceptions about QI activities, including them being the sole responsibility of one individual (generally the practice manager), the failure of clinical audits to deliver change, and lack of patient involvement, were widespread, according to the participants’ views. Some effort could be made to structure QI activities effectively (including clinical audits) within general dental practices. Furthermore, the challenges recognized should be considered while developing QI strategies for general dental practices. This study is part of the DELIVER project, which has a further study planned in which patient feedback will be used to investigate whether and how this influences the quality in dental practices.

## Authors’ Contributions

C. Lin, contributed to data collection, analysis, and interpretation, collectively contributed to data interpretation, drafted and critically revised the manuscript; V. Fehrer, contributed to data collection, analysis, and interpretation, collectively contributed to the data interpretation, drafted and critically revised the manuscript; L. O’Malley, M. Byrne, contributed to data interpretation, collectively contributed to data interpretation, critically revised the manuscript; W. Thompson, collectively contributed to data interpretation, critically revised the manuscript; S. Listl, contributed to conception, collectively contributed to data interpretation, critically revised the manuscript; M. Lorenz, contributed to conception and design, collectively contributed to data interpretation, critically revised the manuscript. All authors gave final approval and agreed to be accountable for all aspects of the work.
